# Oligometastases in prostate cancer: Metabolic response in follow-up PSMA-PET-CTs after hypofractionated IGRT

**DOI:** 10.1007/s00066-017-1239-1

**Published:** 2017-11-27

**Authors:** René Baumann, Mark Koncz, Ulf Luetzen, Fabian Krause, Juergen Dunst

**Affiliations:** 1 Dept. of Radiation Oncology, Christian-Albrechts-University Kiel, Arnold-Heller-Str. 3, 24105 Kiel, Germany; 2 Dept. of Nuclear Medicine, Christian-Albrechts-University Kiel, Kiel, Germany

**Keywords:** Prostate cancer, Oligometastases, PSMA PET, Image-guided radiotherapy, Metabolic response, Prostatakarzinom, Oligometastasen, PSMA-PET, Bildgeführte Strahlentherapie, Metabolisches Ansprechen

## Abstract

**Background:**

Prostate-specific membrane antigen positron emission tomography/computed tomography (PSMAPET/CT) is a new and evolving diagnostic method in prostate cancer with special impact on treatment planning in image-guided radiotherapy (IGRT). Initial results of metabolic response in repeated PSMA PET/CTs after hypofractionated IGRT for metastatic lesions are reported here.

**Materials and methods:**

Of 280 patients investigated with ^68^Ga-PSMA PET/CT in the period from 01/2014 through 12/2016 in the authors’ department, patients were selected according to the following criteria: oligometastatic disease at initial PSMA PET/CT defined as not more than five metastatic lesions, hypofractionated IGRT to all lesions, no systemic therapy in the last 6 months and during follow-up, and at least one follow-up PSMA PET after radiotherapy. Radiotherapy was administered to all PSMA PET-detected lesions (CTV = PET-GTV + 1 to 2 mm), mostly with 35 Gy in five fractions (one lesion with four fractions of 7 Gy due to dose constraints, two lymph nodes with 50 Gy in 25 fractions to an extended volume plus a boost of 7 Gy × 2 to the PET-positive volume). Metabolic response of irradiated lesions was evaluated on repeated PSMA PET/CTs according to PERCIST criteria. Five patients with a total number of 12 PSMA PETs matched the criteria. Patients received radiotherapy to all PET-positive lesions and had at least one (in one case three) follow-up PSMA PET examinations after radiotherapy with an interval to the first PET of 2–15 months; the median follow-up for all patients was 11 months.

**Results:**

The mean prostate-specific antigen (PSA) values at the time of examination were 8.9 ± 8.5 ng/ml (median 3.3 ng/ml, range 0.17–21.8 ng/ml). A total number of 18 metastatic deposits were detected. The PET-positive tumor volume was 5.9 ± 13.3 cm^3^ (median 1.25 cm^3^). The mean standardized uptake value (mean SUV_max_) of the 18 metastatic lesions decreased from 19.9 ± 23.3 (mean ± SD) prior to RT to 5.4 ± 4.6 at post-radiotherapy PSMA PET/CT. Using PERCIST criteria, 14 lesions (78%) showed a metabolic response in PSMA PET with a reduction of SUV of at least 30%, as well as a significant decrease in lesion size; in seven of these lesions, no uptake of ^68^Ga-PSMA was detectable. In follow-up PET scans, only two lesions showed metabolic progression with an increase in SUV_max_ yielding a local progression-free survival of 88% after 1 year. There was a correlation between the time interval after radiotherapy (median 3 months, range 1–9 months) and response (*p* = 0.04) with better metabolic response after longer follow-up.

**Conclusions:**

Preliminary results of this study show high metabolic response rates of PSMA PET-positive metastatic lesions after hypofractionated radiotherapy in follow-up PSMA PET with promising local control rates. An interval of several months may be required to fully estimate the efficacy of radiotherapy in control PSMA PET.

## Zusammenfassung

**Hintergrund:**

Die PSMA-Positronenemissionstomographie/Computertomographie (PET/CT; *PSMA:* prostataspezifisches Membranantigen) ist eine neue und vielversprechende diagnostische Methode bei Prostatakarzinom mit besonderem Einfluss auf die Planung der bildgeführten Strahlentherapie (IGRT). Erste Ergebnisse bezüglich des metabolischen Ansprechens bei wiederholter PSMA-PET/CT nach hypofraktionierter IGRT von Oligometastasen werden im vorliegenden Beitrag dargestellt.

**Material und Methoden:**

In der Zeit von Januar 2014 bis Dezember 2016 wurden an unserer Klinik insgesamt 280 Patienten mit ^68^Ga-PSMA-PET/CT untersucht. Davon wurden Patienten ausgewählt, die die folgenden Kriterien erfüllten: Oligometastasierung in der ersten PSMA-PET/CT mit maximal 5 Herden, hoch dosierte hypofraktionierte IGRT für alle Herde, keine medikamentöse antihormonelle oder zytostatische Therapie in den letzten 6 Monaten und im Follow-up, mindestens eine PSMA-PET/CT zur Kontrolle des Ansprechens der Herde vorhanden. Alle in der PSMA-PET detektierten Herde (CTV = PET-GTV plus 1–2 mm) wurden mit der Strahlentherapie behandelt, überwiegend mit 35 Gy in 5 Fraktionen (ein Herd mit 4 Fraktionen à 7 Gy wegen einer Dosisbeschränkung, 2 Lymphknoten mit 50 Gy in 25 Fraktionen auf ein erweitertes Volumen plus Boost von 7 Gy × 2 auf das PET-positive Volumen). Das metabolische Ansprechen bestrahlter Herde wurde in wiederholten PSMA-PET/CT gemäß den PERCIST-Kriterien untersucht. Fünf Patienten mit 12 PSMA-PET-Untersuchungen erfüllten die Kriterien. Bei diesen Patienten wurden alle PET-positiven Herde mit hypofraktionierter IGRT auf das PET-positive Volumen behandelt. Sie erhielten mindestens eine (in einem Fall 3) Follow-up-PSMA-PET-Untersuchungen nach Strahlentherapie mit einem Intervall zur ersten PET von 2 bis 15 Monaten; die Follow-up-Dauer aller Patienten betrug im Median 11 Monate.

**Ergebnisse:**

Der mittlere Wert des prostataspezifischen Antigens (PSA) zum Zeitpunkt der ersten PSMA-PET/CT betrug 8,9 ± 8,5 ng/ml (Median 3,3 ng/ml, Spanne 0,17–21,8 ng/ml). Insgesamt wurde 18 Metastasen detektiert. Das PET-positive Tumorvolumen betrug 5,9 ± 13,3 cm^3^ (Median 1,25 cm^3^).

Der mittlere „standardized uptake value“ (SUV_max_) der 18 Metastasen sank von 19,9 ± 23,3 (Mittelwert ± Standardabweichung) vor der Radiotherapie auf 5,4 ± 4,6 in der PSMA-PET/CT nach Radiotherapie.

Gemäß den PERCIST-Kriterien zeigten 14 Metastasen (78 %) eine metabolische Remission in der PSMA-PET mit Reduktion des SUV_max_ von mindestens 30 % und einer signifikanten Größenabnahme; in 7 dieser Metastasen war eine ^68^Ga-PSMA-Aufnahme nicht mehr nachweisbar. Follow-up-PET-Untersuchungen ergaben nur für 2 Herde eine metabolische Progression mit SUV_max_-Anstieg; das entspricht einer lokalen Progressionsfreiheit von 88 % nach einem Jahr. Es bestand ein signifikanter Zusammenhang zwischen dem Zeitintervall nach Radiotherapie (Median 3 Monate, Spanne 1–9 Monate) und dem Ansprechen (*p* = 0,04) mit besserem metabolischem Ansprechen bei längerem Intervall.

**Schlussfolgerung:**

Diese vorläufigen Ergebnisse zeigen eine hohe metabolische Ansprechrate von PSMA-PET-positiven metastatischen Herden nach hypofraktionierter Strahlentherapie in der Follow-up-PET. Die Raten der lokalen Kontrolle sind vielversprechend. Ein Intervall von mehreren Monaten erscheint notwendig, um die Wirksamkeit der Strahlentherapie in der Kontroll-PSMA-PET umfassend beurteilen zu können.

## Introduction

Local treatment of metastatic lesions in oligometastatic cancers is a new and promising approach for many types of cancers, namely breast, colorectal, non-small cell lung and prostate cancers. Moreover, retrospective studies with stereotactic body radiotherapy to metastatic sites have reported promising results, suggesting that local therapy might be able to improve time to progression and delay the initiation of systemic therapy in oligometastatic prostate cancer [13, 17, 21]. Randomized studies in prostate cancer are ongoing.

Prostate-specific membrane antigen positron emission tomography (PSMA PET) is a new and rapidly evolving diagnostic tool for the detection of metastases in prostate cancer. PSMA is expressed in normal prostate cells, but its expression is increased in prostate cancer cells by a factor of 100- to 1000-fold [4, 22]. Expression increases with cancer stage. Thus, PSMA is an attractive tool for imaging. Initial results with an In-labelled antibody were hindered by low affinity, but recently, a more specific binding antibody labelled with Ga-68 was developed [1, 2, 20]. Several groups have reported high sensitivity and specificity rates of more than 80% up to 97% for both patient and lesion analysis [3, 5–11, 14–16, 18, 19, 23]. Lesion detection was possible even in cases of very low PSA values with a detection rate of about 40% in patients with PSA values below 0.5 ng/ml. Thus, PSMA PET offers a new tool for the diagnosis of oligometastatic prostate cancer in a very early course of metastatic disease. Moreover, precise localization of metastatic deposits can be used as a basis for image-guided radiotherapy (IGRT) to detected sites. We report initial results with special emphasis on response in follow-up PET/computed tomography (CT) after high-dose IGRT of PSMA PET-detected lesions.

## Materials and methods

### Patients

Between January 2014 and December 2016, 280 patients with prostate cancer received at least one PSMA PET/CT examination in the authors’ department. A total of 33 patients had repeated PSMA PET examinations during follow-up; the reason for performing repeated PET investigation was persisting or newly increased serum PSA level. Out of this group, patients were selected according to the following criteria: oligometastatic disease at initial PSMA PET/CT defined as not more than five metastatic lesions, hypofractionated IGRT to all PSMA PET-detected lesions, no systemic therapy in the previous 6 months and during the entire follow-up period and at least one follow-up PSMA PET after radiotherapy.

### PSMA PET/CT

^68^Ga ws obtained from a ^68^Ge/^68^Ga generator, labelled with the PSMA-11-precursor (ABX, Radeberg, Germany) and administered via an intravenous bolus injection (mean of 109.78 MBq, range 73–148 MBq). For the examination of the patients of this study, a cross-calibrated dedicated PET/CT system, Biograph mCT 40 (Siemens, Erlangen, Germany) was used and a standard acquisition protocol with a whole-body scan (skull base to upper thighs) starting between 58 and 95 min (mean: 70.6 min.) p. i. and three examinations were additionally performed between 120 and 150 min (mean:130 min.) p. i. as part-body scans. Image data analysis was performed using commercially available software (Siemens, Erlangen, Germany). For the calculation of the SUV_max_, the volume-of-interest technique was used around the areas with an increased tracer uptake in multiplanar imaging.

PSMA PET during follow-up was not performed routinely, but only in case of repeated increase in serum PSA.

### Radiotherapy

Treatment decisions were based on multidisciplinary case discussions and the decision on local treatment was made with the objective to avoid and postpone systemic therapy. Radiotherapy was administered to all PSMA PET-detected lesions. The clinical target volume (CTV) was defined as the gross tumor volume (GTV) visible on PSMA PET (volume 50% of SUV_max_) plus a safety margin of 2 mm (CTV = PET-GTV + 2 mm; safety margin of 1 mm in selected anatomic sites, e. g. vertebrae). All patients were treated at a Varian TrueBeam STX with daily IGRT and (in selected cases) use of the stereotactic Novalis STX system using standard immobilization devices. The standard fractionation regimen for oligometastatic disease was five fractions with a 7 Gy fraction dose. In one patient, two PET-positive lymph node metastases were treated with 50 Gy in conventional fractionation to an extended volume covering the node areas followed by a boost of 7 Gy × 2 to the PET-positive lymph nodes. In one patient, a single PET-positive lymph node adjacent to the esophagus was treated with four fractions of 7 Gy due to dose constraints. Treatment was given on consecutive working days without interruptions except at weekends.

### Response evaluation

For response evaluation, follow-up PSMA PETs were compared to the initial PSMA PET prior to radiotherapy. For the evaluation of metabolic response, the standard RECIST criteria are not useful. For fluorodeoxyglucose (FDG)-PET, a modification to the RECIST criteria, the PERCIST criteria, was recommended [24]. Although such specific response criteria have not yet been published for PSMA PET, PERCIST offers the best system currently available for response evaluation of PET data and was therefore used. Thus, metabolic response was defined as a reduction in SUV_max_ of at least 30% with no increase in lesion size.

### Statistical analysis

Data were analyzed using SPSS with descriptive statistics and univariate analysis. Multivariate analysis was not performed due to sample size.

The study was approved by the ethics committee of the Medical Faculty of the Christian-Albrecht University Kiel.

## Results

### Patients and treatment characteristics

Five patients fulfilled the criteria; all patients had oligometastatic disease after successful primary treatment (Table 1). None of the patients had received systemic treatment within the previous 6 months or during follow-up after radiotherapy. Four patients had one and one patient had three follow-up PSMA PET/CTs with intervals to the first PET of 2–15 months. The median follow-up of the five patients was 11 months. The mean PSA value (mean ± 1 SD) at the time of the first PET was 8.9 ± 8.5 ng/ml (median 3.3 ng/ml, range 0.17–21.8 ng/ml). At follow-up PSMA PET, mean PSA values were 21.8 ± 25.8 ng/ml (median 8.7 ng/ml, range 0.62–77.8 ng/ml). This increase in PSA was due to the selection bias (repeated PET only in the case of a repeated rise in serum PSA). All patients had new lesions on follow-up PET/CT. This analysis focuses on formerly detected and irradiated lesions and the response of these lesions in the follow-up PET.

**Table 1:** Characteristics of patients and treated metastatic lesions

Patient ID/age (years)	Characteristics of initial tumor and primary treatment	Characteristics of metastatic lesion in first PSMA PET				Interval between radiotherapy and second PSMA PET (months)	Metabolic response
		Site	Volume (cm^3^)	SUVmax	IGRT fractionation		
HD, 66	pT3b R1 pN1 Gleason 9 Radical prostatectomy	TH 7 Right humerus rib	3.5	61.47	7.0 Gy × 5	9.8	mR
			3.2	90.24	7.0 Gy × 5	9.8	mR
			5.1	23.90	7.0 Gy × 5	9.8	mR
RB, 72	pT3 R1 pN1 Gleason 9 Radical prostatectomy + XRT to prostate bed + 6 months ADT	Rib	0.1	3.17	7.0 Gy × 5	5.0	mR
		Rib	1.9	5.18	7.0 Gy × 5	5.0	mR
		Pelvic node	1.1	7.32	2.0 Gy × 25 + 7. Gy × 2	2.9	mR
		Pelvic node	0.8	3.98	2.0 Gy × 25 + 7. Gy × 2	2.9	mR
		Th 9	1.2	6.78	7.0 Gy x 5	4.2	mR
		Mediastinal node	1.5	5.33	7.0 Gy × 4	2.2	Progressive
MH, 64	pT3 R1 pN2 Gleason 9 Radical prostatectomy + XRT to prostate bed + 6 months ADT + 4 months dendritic cells	Iliac bone	14.8	18.59	7.0 Gy × 5	3.7	Stable
		Iliac bone	1.3	10.76	7.0 Gy × 5	3.7	mR
		L 3	1.6	6.27	7.0 Gy × 5	3.7	mR
		Scapula	0.5	3.99	7.0 Gy × 5	3.7	Progressive
		Mediastinal node	6.6	9.28	7.0 Gy × 5	3	Stable
MP, 77	pT2 pN1 Radical prostatectomy	Rib	2.3	14.26	7.0 Gy × 5	15	mR
		Sacrum	1.6	38.25	7.0 Gy × 5	15	mR
		L4	58.8	44.57	7.0 Gy × 5	15	mR
KT, 78	Radical prostatectomy, salvage-XRT to prostatic bed + ADT	Rib	0.8	4.25	7.0 Gy × 5	1.9	mR

*ADT* androgen-deprivation therapy, *TH* thoracic vertebra, *L* lumbar vertebra, *mR* metabolic response

A total number of 18 metastatic deposits were detected at the first PSMA PET prior to radiotherapy. The PET-positive tumor volume was 5.9 ± 13.3 cm^3^ (median 1.25 cm^3^, range 0.1–58.8 cm^3^). The mean SUV_max_ value of all 18 metastatic lesions was 19.9 ± 23.3.

In all, 14 metastatic lesions were bony metastases; 5 metastases were located in the spine, five in the ribs and the others in pelvic bones (*N* = 2), the scapula and the humeral bone (each *N* = 1). All lesions were treated with a hypofrationated regimen (35 Gy in five fractions). Four metastatic lesions were lymph node metastases; they were treated either with 50 Gy in conventional fractionation followed by a boost of 7 Gy × 2 (in a patient who had previously undergone salvage radiotherapy to the prostatic bed) or with four (in one case) or five fractions (in another case) with 7 Gy. All patients completed radiation treatment without interruptions or severe adverse events. No acute toxicity and no late side effects were observed.

### Metabolic response

The mean interval between the end of radiotherapy and the follow-up PET/CT was 3 months (range 1.9–15 months). The mean SUV_max_ value of all 18 metastatic lesions decreased from 19.9 ± 23.3 prior to RT to 5.4 ± 4.6 at post-radiotherapy PSMA PET/CT. Of 18 lesions, 14 (78%) showed a metabolic response on PSMA PET with a reduction of SUV_max_ of at least 30%, as well as a significant decrease in lesion size; in seven of these lesions (39%), no uptake of ^68^Ga-PSMA was detectable with SUV values below the threshold on follow-up PET/CT. Two lesions showed stable disease on follow-up PET/CT and two lesions showed progression with an increase in SUV_max_ > 25% (one bone metastasis after five fractions of 7 Gy and a paraesophageal lymph node that was treated with only four fractions to 28 Gy).

### Association between time interval and metabolic response

**Fig. 1:**
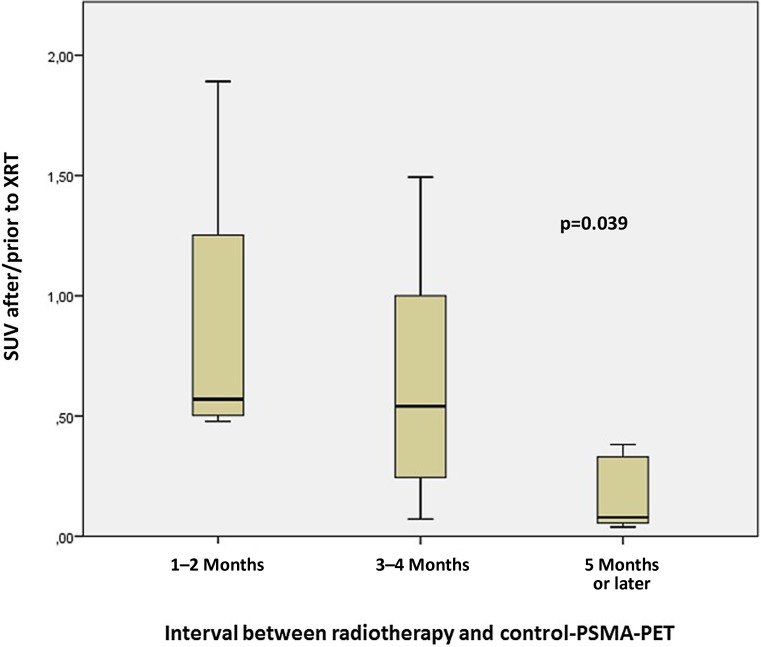
Impact of interval between radiotherapy and follow-up PSMA PET/CT on metabolic response (change in SUV_max_). Response improved significantly with longer follow-up

There was a correlation between the time interval after radiotherapy and response (*p* = 0.04; Fig. 1). The decrease in SUV_max_ was more pronounced after longer follow-up. The metabolic response rate was 100% if the control PSMA PET was performed after an interval of 5 months or more.

### Recurrences

New metastatic lesions were detected on all follow-up PSMA PET/CTs. All lesions were outside the irradiated areas. No progression in the irradiation volume had been observed hitherto after a metabolic response. Local progression-free survival (using metabolic response as a parameter for local control) at the irradiated sites was 88% after 1 year; for lesions treated with five fractions of 7 Gy to a total dose of 35 Gy, the local control rate was 100% after 1 year.

## Discussion

To the authors’ knowledge, this is the first report on response evaluation of metastatic lesions with follow-up PSMA PET/CT after high-dose IGRT for PSMA PET-detected oligometastases in prostate cancer. The data are preliminary and there are several limitations. First of all, the patients in this analysis were highly selected with the objective to investigate the efficacy of radiotherapy as the sole treatment in patients that were naïve with regard to systemic therapy. Moreover, patients had stable or rising PSA levels at the time when PSMA PET was repeated; this bias was due to the fact that an indication for a second or third PSMA PET was given only in cases of rising PSA. Therefore, the response of serum PSA could not be evaluated in this investigation. Thirdly, all patients had new lesions, as expected due to the PSA increase at follow-up PET. Fourthly, follow-up was relatively short at a median of 11 months. Nevertheless, there are some facts that are interesting for further studies.

In this investigation, the detected and treated lesions were small with median and mean volumes of 1.25 cm^3^ and 5.9 cm^3^, respectively. According to data from other investigations, the detection rate of PSMA PET is clearly higher than that of all other currently available imaging methods [12, 19]. It is very likely that at least a relevant number of these lesions would not have been detected with routine staging procedures. Thus, PSMA PET seems to be a powerful tool for treatment planning in oligometastatic prostate cancer patients that are scheduled for local ablative therapies. A major issue for patients in whom PSMA PET is used for radiotherapy planning concerns the question of how to define the target volume for radiotherapy. This is important especially in patients scheduled for high-precision radiotherapy techniques. With regard to the primary tumor in the prostate, PSMA PET seems to overestimate the tumor volume as compared with magnetic resonance imaging (MRI) [25]. The PET-positive volume was used for target definition and additional imaging methods were not routinely used for better definition of size of the treated lesions. Overestimation of the target volume cannot be ruled out, but is probably not an issue in a single, anatomically well-defined lymph node. However, target definition may be more difficult in bony structures. PSMA PET may underestimate, at least theoretically, a microscopic extension of tumor in the bone marrow, e. g. in the iliac bone or a vertebra. Therefore, a small safety margin to cover such microscopic extension was used. The preliminary results from this investigation suggest that this approach can be used in daily clinical practice.

Secondly, high metabolic response rates of PSMA PET-detected lesions after hypofractionated radiotherapy were observed. Altogether, 39% of all lesions were no longer detectable after radiosurgery, and a further 39% showed a metabolic partial response according to PERCIST criteria. The interval between the end of radiotherapy and follow-up PSMA PET was relatively short (median 3 months) and might not have been sufficient to estimate the full efficacy of radiotherapy. The best response was seen in the patient with the longest interval between radiotherapy and re-investigation, and a significant association between the follow-up interval and metabolic response was found. These results suggest that an interval of 6 months or more may be required to fully estimate the efficacy of radiotherapy in PSMA PET imaging.

Last but not least, hypofractionated IGRT with five fractions of 7 Gy is a treatment regimen with proven efficacy in the primary treatment of prostate cancer [15]. This regimen has also been used by other groups to treat metastases [13]. The results reported here confirm that this regimen is feasible even for metastatic sites with high efficacy and good tolerance.

In summary, the preliminary results reported here suggest that PSMA PET-detected metastatic lesions can be effectively treated with high-precision radiotherapy to the PSMA PET-positive tumor volume. The course of metabolic response in follow-up PSMA PET/CT requires further investigation. If PSMA PET is used for response evaluation, an interval of several months after radiotherapy may be required to fully estimate the efficacy of radiotherapy.
